# circ-ANXA7 facilitates lung adenocarcinoma progression via miR-331/LAD1 axis

**DOI:** 10.1186/s12935-021-01791-5

**Published:** 2021-02-03

**Authors:** Yu Wang

**Affiliations:** Department of Medical Laboratory, Zhumadian City Central Hospital, No. 747 Zhonghua Avenue, Yicheng District, Zhumadian, 463000 Henan China

**Keywords:** Lung adenocarcinoma, circ-ANXA7, Proliferation, Invasion, miR-331, LAD1

## Abstract

**Background:**

Lung adenocarcinoma (LUAD) is the most common histological subtype of lung cancer, with a poor prognosis. The roles of circular RNAs (circRNAs) in tumors have been initially clarified. In this study, we probed into the functions and underlying molecular mechanisms of circ-ANXA7 in LUAD.

**Methods:**

According to circRNA microarray analysis based on 40 pairs of LUAD tissues and non-tumor tissues, a novel circ-ANXA7 was up-regulated in LUAD, which was verified in LUAD tissues and cells by RT-qPCR. Correlation between its expression and clinical features of LUAD was analyzed. When transfected with sh-circ-ANXA7, proliferation, invasion, and migration of LUAD cells were determined by a series of functional assays. Furthermore, tumor growth was investigated in nude mice injected with sh-circ-ANXA7. Dual luciferase report and gain and loss assays were used to confirm the relationships between circ-ANXA7 and miR-331, miR-331 and LAD1.

**Results:**

circ-ANXA7 was up-regulated in LUAD tissues and cells. Its high expression promoted proliferation, migration, and invasion of LUAD cells as well as tumor growth. High circ-ANXA7 expression usually predicted a poorer prognosis for LUAD patients. Furthermore, circ-ANXA7 could accelerate proliferation and invasion of LUAD cells by targeting miR-331. miR-331 directly bound to the 3′-UTR of LAD1. LAD1 induced proliferation and invasion of LUAD cells, which was reversed after co-transfection with circ-ANXA7 knockdown. LAD1 expression could be an independent prognostic marker for LUAD by univariate and multivariate analysis.

**Conclusions:**

Our research identified a novel circ-ANXA7 for LUAD, which could facilitate proliferation, migration, and invasion of LUAD cells by miR-331/ LAD1 axis. circ-ANXA7 could become a promising prognosis and treatment target for LUAD.

## Background

Lung cancer is one of the most frequently diagnosed malignancies worldwide, with extremely high morbidity and mortality [1]. Non-small cell lung cancer (NSCLC) is the main histological subtype of lung cancer (approximately 85%), composed of lung adenocarcinoma (LUAD) and lung squamous cell carcinoma (LUSC) [2]. LUAD accounts for about 85% of all cases of lung cancer, especially among women and young people [3]. Despite the improvement in early diagnosis and therapy, the 5-year survival rate of LUAD is still < 20% [4]. Thus, it is of importance to probe into the molecular mechanisms of LUAD.

Circular RNA (circRNA), an endogenous non-coding RNA, possesses a covalent closed-loop structure that endows resistance to exonuclease [5–7]. In the past few years, numerous circRNAs have been identified in various diseases, especially cancers [8–10]. The role of circRNAs in tumor progression reveals the diversity of cancers [11]. Growing evidence suggests that circRNA can act as a miRNA sponge to mediate the expression of target mRNA [12]. Recently, it has been identified that several circRNAs participate in LUAD progression, such as circ-000032 [13], circ-PVT1 [14] and circ-ABCC4 [15]. Nevertheless, the function of most circRNAs in LUAD remains unknown.

In this study, circRNAs between LUAD and non-cancer tissues were characterized by circRNA microarray and quantitative reverse transcription PCR (qRT-PCR). We found that circ-ANXA7 was up-regulated in LUAD tissues, and its high expression predicted a poor prognosis. A series of functional studies demonstrated that circ-ANXA7 accelerated proliferation and invasion of LUAD cells as well as tumor growth. Mechanistically, circ-ANXA7 could enhance the expression of LAD1 by acting as a sponge of miR-331. As previous studies, miR-331 has been identified to be downregulated in NSCLC tissues [16]. Its overexpression can suppress proliferation, migration, and metastasis of NSCLC cells [17]. miR-331 expression is related to clinicopathological features of NSCLC, which may become an independent prognostic indicator for NSCLC patients [16]. Furthermore, it has been found that LAD1 is overexpressed in LUAD tissues than normal tissues [17]. However, its regulatory mechanisms with miRNAs and roles in LUAD progression remain unclear. Thus, our results indicated that circ-ANXA7 was involved in the progression of LUAD and could be used as an underlying prognostic factor.

## Materials and methods

### Tissue specimens

Totally, 40 pairs of fresh tumor and normal adjacent tissue specimens were gathered from LUAD patients during surgery in the Zhumadian Central Hospital between 2012 and 2013. Paired histologically-normal adjacent lung tissues were gathered from the same patient at tumor resection. These specimens were then immediately frozen in liquid nitrogen and stored at − 80 °C. None of them experienced radiotherapy and chemotherapy before surgery. Furthermore, a total of 90 NSCLC TMA samples were collected. All patients were diagnosed in line with the 7th edition of IASLC stage project [18]. All participants provided written informed consent. This study gained the approval of the Ethics Committee of Zhumadian Central Hospital (2012019).

### Microarray analysis

Microarray analysis of 40 pairs of fresh tumor and non-tumor tissue specimens was achieved by Genechem Co., LTD. (Shanghai, China). Total RNA was isolated from tissues utilizing TRIzol reagent (Invitrogen, Carlsbad, CA, USA), which was then stored at − 80 °C. The procedures of miRNA and mRNA microarray analysis was as previously described [19]. For circRNA microarray analysis, total RNA was digested with RNase R (Epicentre; Illumina, Inc., San Diego, CA, USA). Linear RNAs were removed and circRNAs were enriched. After amplification, the enriched circRNAs were transcribed into fluorescent cRNA. Arraystar Human circRNA Microarray analysis (Arraystar Inc., Rockville, MD, USA) was then presented. Differentially expressed circRNAs between LUAD and normal groups were identified with the threshold of adjusted P < 0.05 and fold change > 2.

### Cell culture

Two human normal bronchial or lung epithelial cells (16HBE and BEAS-2B) and five human LUAD cell lines including NCI-H1299, A549, SPC-A1, PC9 and NCI-H1650 were purchased from Shanghai Cell Bank, Chinese Academy of Sciences (China), which were cultured in 1640 medium (Hyclone, Beijing, China) plus 10% fetal bovine serum (FBS; gibco, California, USA) at 37 °C and 5% CO_2_.

### qRT-PCR

Total RNA was extracted from tissues or cells via RNA extraction kit (ThermoFisher, Beijing, China). mRNA and circRNA were reverse transcribed using Prime Script TMRT Master Mix kit (Takara, Beijing, China). miRNA reverse transcription was carried out using MiR-XTM miRNA First-Strand Synthesis kit (Takara). PCR was performed using SYBR®Premix Ex Taq™ kit (Takara). GAPDH was an internal reference. circ-ANXA7: 5′-GCTATCCCCCAACAGGCTAC-3′ (forward), 5′-CCTGGTGGGACTCCAAATC-3′ (reverse); GAPDH: 5′-AGAAGGCTGGGGCTCATTTG-3′ (forward), 5′-AGGGGCCATCCACAGTCTTC-3′ (reverse). The relative expression was quantified with 2^−ΔΔCt^ method.

Primer sequences.

### Transfection

Cells were covered onto the 6-well plate. sh-circ-ANXA7 (RiboBio Co., LTD, Guangzhou, China), pcDNA3.1-ANXA7, miR-331 mimics, miR-331 inhibitors, pcDNA3.1-LAD1 and corresponding negative control (NC) were transfected into cells via Lipofectamine 2000 (Invitrogen, Carlsbad, California, USA). After 24 h, transfection effect was verified by qRT-PCR.

### Cell counting kit-8 (CCK‐8) assay

The cells in the logarithmic growth phase were digested with 0.25% trypsin for 3 min and prepared into a single cell suspension. After being diluted with the culture medium, the cells were counted on a hemocytometer under a microscope. The counting formula was as follows: number of cells/mL = (number of cells in each large cell) × 10^4^ × dilution factor. Cells were added to a 96-well plate at 37 °C 5% CO_2_ incubator for 6 h (1000 cells/well). After the cells adhered to the wall, it was counted as 0 h. CCK8 experiments (Dojindo, Japan) were performed at 0 h, 24 h, 48 h, 72 h and 96 h time points. Then, 10% CCK-8 reagent was added to the plate. After incubation for 1–2 h, a microplate reader was used to detect cell viability. The detection wavelength was set to 450 nm.

### Colony formation assay

Cells were seeded into 6-well plates (3000/well). After 1 week, the cells were fixed with 600 µL methanol for 30 min. The cells were stained with 600 µL 0.1% crystal violet for 20 min. 500 µL 33% glacial acetic acid was added to each well to wash away crystal violet. Crystal violet solution was added into 96-well plate. The absorbance value at 570 nm was detected.

### 5-Ethynyl-2′-deoxyuridine (EdU) staining

Cell viability was measured by the Cell-Light™ EdU kit (RIBOBIO, Guangzhou, China). LUAD cells were seed onto 96-well plate. 100 µL 50 µM EdU medium was added to the plate for 2 h. Complete medium without EdU was used as a negative control. After discarding the culture solution, cells were incubated by 100 µL cell fixation solution for 20 min at room temperature, followed by 2 mg/mL glycine for 10 min. Following discarding the supernatant, 100 µL PBS buffer containing 0.5 % TritonX-100 was used to permeabilize the cells. Then, cells were incubated with 100 µL 1×Apollo® staining reaction solution and 1×DAPI reaction solution at room temperature in the dark for 30 min. The results were observed under a fluorescence microscope (Olympus, Japan).

### Transwell assay

Matrigel (BD, New Jersey, USA) was spread on the upper layer of transwell chamber (Corning, Shanghai, China) at 37 ℃ overnight. 3 × 10^4^ cells were added to the upper layer and 800 µL complete medium plus 20% FBS was added to the lower layer. After 48 h, transwell chamber was harvested. The cells in the lower layer were fixed with 4% paraformaldehyde (Biosharp, Shanghai, China) for 20 min. Then, the cells were stained by 0.1% crystal violet staining solution (Beyotime) for 15 min. After washing twice with ddH2O, the cells and Matrigel in the upper layer were wiped off. Three areas were randomly selected to take pictures under a microscope (400×).

### Wound healing assay

First, a horizontal line was drawn on the back of the six-well plate. The cells were seeded in the plate and cultured overnight. When the cells covered the plate surface, 200 µL pipette tip was used to scratch the six-well plate. After washing three times with PBS, the cells were incubated with serum-free medium at 37 °C and 5% CO_2_. Photos were taken at 0 h and 24 h.

### Animal experiments

Twelve 5-week-old nude male BALB/c mice were purchased from Shanghai Laboratory Animal Research Center (China). All mice were housed under independent ventilation cages (IVCs), which were randomly divided into two groups. 3 × 10^6^ PC9-NC or PC9-sh-circ-ANXA7 cells were subcutaneously injected into the single flank of mice to establish LUAD model. After mice were euthanized by excess sodium pentobarbital, tumor weight was detected. Furthermore, tumor volume was measured each week. This animal experiment was presented in strictly line with the recommendations in the Guide for the Care and Use of Laboratory Animals of the National Institutes of Health, which was approved by Zhumadian Central Hospital Animal Ethics Research Board (2018046).

### Haematoxylin and eosin (H&E) staining and immunohistochemistry

The tumor tissues were fixed with 4% formaldehyde solution, embedded in paraffin, and cut into 4 µm thickness. Subsequently, H&E staining and immunohistochemical staining were presented. For H&E staining, the sections were stained with hematoxylin for 5 min and stained with eosin staining solution for 3 min. For immunohistochemical staining, the sections were incubated with mouse anti-human Ki67 monoclonal antibody (ab245113, Abcam, Cambridge Science Park, CK) at 4 °C overnight. The sections were then incubated with HRP-labeled Goat Anti-Mouse IgG (ab150113, Abcam) at 37 °C for 20 min. Hematoxylin staining solution was used to stain the nuclei. After ethanol dehydration and xylene transparency, the sections were sealed with neutral gum. The stained sections were observed under an optical microscope (400×).

### Dual luciferase report

LUAD cells or 293T cells (Chinese Academy of Sciences) were co-transfected with 250 ng pmiR-GLO-NC/circ-ANXA7-wt/circ-ANXA7-mut/LAD1-wt or LAD1-mut (Sangon Biotech, China). Furthermore, the cells were co-transfected with pPG-miR-NC or miR-331. Dual-luciferase reporter assay kit was utilized to evaluate luciferase activity. Relative luciferase activity was normalized to Renilla luciferase activity.

### Western blot

Cells were lysed by RIPA buffer (Sigma, New York, USA), followed by 12,000 rpm centrifugation at 4 ℃ for 15 min. Then, the supernatant was collected and total protein concentration was determined by BCA kit (ThermoFisher). Samples were separated by polyacrylamide gel electrophoresis (SDS-PAGE) and transferred into PVDC membrane (Millipore, Massachusetts, USA) for 2 h. Then, the membrane was blocked with TBS buffer with 3% BSA for 1 h at room temperature, which was incubated with anti-LAD1 antibody (1:1000; ab246885, Abcam), anti-ANXA7 (1:1000; ab197586, Abcam) and GAPDH (1:1000; ab8245, Abcam) at 4 ℃ overnight and Goat Anti-Rabbit IgG (1:3000; ab150077, Abcam) at room temperature for 2 h. Following the instructions of the Enhanced chemiluminescence kit, the membrane was illuminated.

### Statistical analyses

Statistical analyses were achieved by R language and GraphPad Prism 8.0 software. Data were expressed as mean ± standard deviation. 40 cases of LUAD patients were separated into high or low circ-ANXA7 groups in accordance to its median expression value. Kaplan–Meier survival analysis was carried out between the two groups, followed by log-rank test. Multivariate regression analysis of circ-ANXA7 expression was achieved following adjusting other prognostic factors including recurrence, lymph metastasis, tumor size, TNM stage, histologic subtype, smoking history, gender, and age. The comparison between two groups was presented by paired student’s t test, while multiple comparisons were presented using one-way ANOVA with Tukey’s post hoc test. P-value < 0.05 was set as a cutoff value.

## Results

### circ-ANXA7, a novel up‐regulated circRNA, is an independent prognostic factor for LUAD

**Fig. 1:**
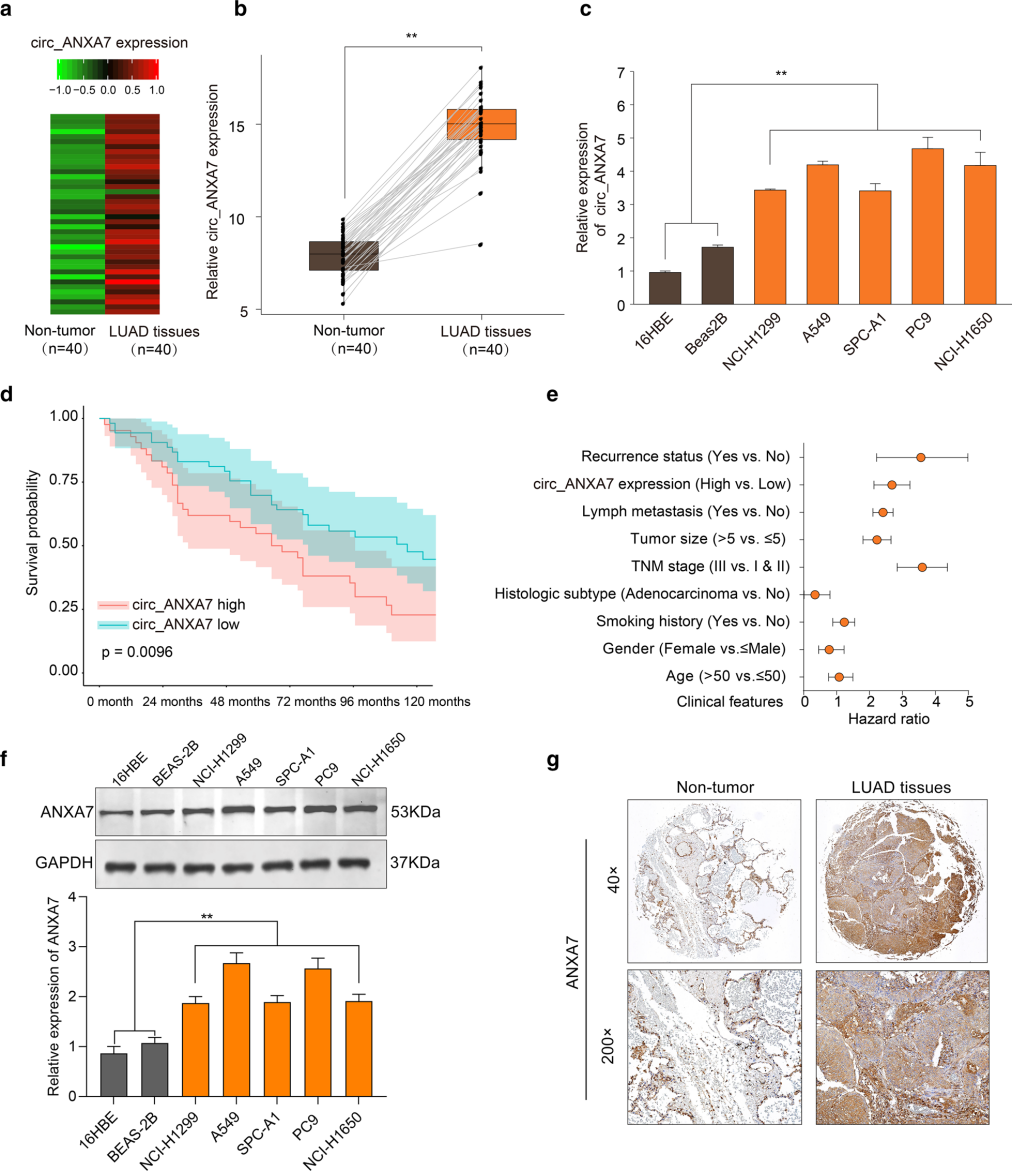
A novel up-regulated circRNA circ-ANXA7 is an independent prognostic factor for LUAD. **a** circ-ANXA7 up-regulation was found between LUAD tissues (n = 40) and non-tumor tissues (n = 40) by microarray analysis. Red: up-regulation and green: down-regulation. **b** Box plots visualizing a higher expression level of circ-ANXA7 in LUAD tissues than non-tumor tissues using qRT-PCR. **c** qRT-PCR was utilized to examine the relative expression levels of circ-ANXA7 between normal epithelial cells and LUAD cells. **d** Overall survival analysis between LUAD patients with high circ-ANXA7 expression and those with its low expression. **e** Multivariate regression analysis of circ-ANXA7 expression after adjusting other prognostic factors. **f** Western blot was presented to examine the expression of ANXA7 protein between normal epithelial cells and LUAD cells. **g** Immunohistochemistry of ANXA7 between adjacent normal tissues and LUAD tissues. Magnification: ×40; ×200. **p < 0.01

Based on our microarray analysis results, circ-ANXA7 was found to be prominently up-regulated in LUAD tissues (n = 40) compared to non-tumor tissues (n = 40). Heat map visualized the expression patterns of circ-ANXA7 between tumor and non-tumor samples (Fig. 1a). qRT-PCR results confirmed its up-regulation in LUAD tissues (Fig. 1b). Also, compared to normal bronchial or lung epithelial cells, circ-ANXA7 expression was distinctly elevated in different LUAD cells (Fig. 1c). Patients with high circ-ANXA7 expression usually indicated a poorer overall survival than those with its low expression (Fig. 1d). After multivariate regression analysis, circ-ANXA7 expression could be an independent prognostic factor LUAD (Fig. 1e). Furthermore, we examined the expression of ANXA7 protein in LUAD. Our data showed that ANXA7 expression was distinctly up-regulated in different LUAD cells compared to normal bronchial or lung epithelial cells (Fig. 1f). From Human Protein Atlas database (https://www.proteinatlas.org/), immunohistochemistry (IHC) of ANXA7 was obtained. As shown in Fig. 1g, its higher expression wad found in LUAD tissues than adjacent normal tissues.

### circ-ANXA7 facilitates proliferation, migration and invasion of LUAD cells

**Fig. 2:**
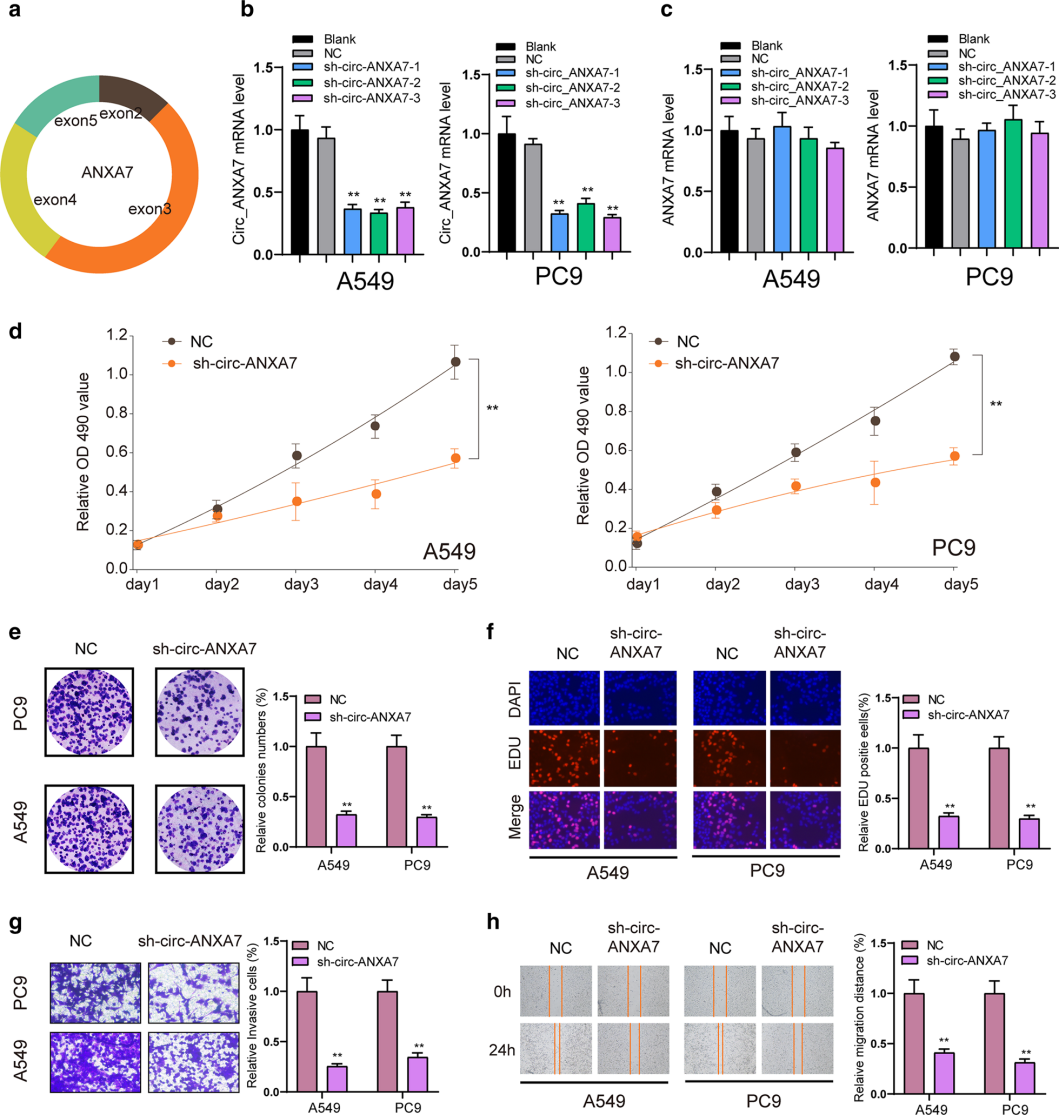
circ-ANXA7 facilitates proliferation and invasion of LUAD cells. **a** Schematic showing the schematic of specific PCR primers for circ-ANXA7. **b**, **c** RT-qPCR was utilized to examine the expression of circ-ANXA7 and ANXA7 in A549 and PC9 LUAD cells transfected by sh-circ-ANXA7. **d**–**f** CCK-8, clone formation and EdU assay results showed that cell viability of LUAD cells was suppressed following transfection with sh-circ-ANXA7 compared to the NC group. **g**, **h** Transwell invasion and wound healing experiments confirmed that LUAD cells transfected with sh-circ-ANXA7 displayed a lower invasion and migration abilities. *p < 0.05; **p < 0.01

Figure 2a shows the schematic of specific PCR primers not convergent primers for specific detection of circ-ANXA7. To further explore the role of circ-ANXA7 in LUAD cells, this study designed and synthesized three shRNAs targeting circ-ANXA7. sh-circ-ANXA7 was transfected into A549 and PC9 cells. qRT-PCR was used to analyze the expression of circ-ANXA7. The results showed that, compared with the NC group, sh-circ-ANXA7 only significantly reduced the expression of circ-ANXA7 (Fig. 2b) without affecting the expression of ANXA7 (Fig. 2c), suggesting the specificity of shRNAs targeting circ-ANXA7. CCK-8, clone formation and EdU assays were used to investigate the proliferation of A549 and PC9 LUAD cells after transfection of sh-circ-ANXA7. In Fig. 2d, 5 days after shRNA transfection, the viability of LUAD cells was distinctly reduced compared to the NC group. As shown in Fig. 2e, sh-circ-ANXA7 remarkably cut down the number of colonies in LUAD cells. Furthermore, EdU assay results showed that relative EdU staining positive cells in the sh-circ-ANXA7 group was prominently reduced (Fig. 2f). This study further investigated the cell migration and invasion ability after down-regulation of circ-ANXA7. Transwell invasion experiments showed that A549 and PC9 cells exhibited lower invasive ability after transfection with sh-circ-ANXA7 than the NC group (Fig. 2g). The wound healing experiment was utilized the migration ability of cells. The results showed that sh-circ-ANXA7 conspicuously inhibited the wound closure (Fig. 2h).

### circ-ANXA7 knockdown suppresses tumor growth in vivo for LUAD

**Fig. 3:**
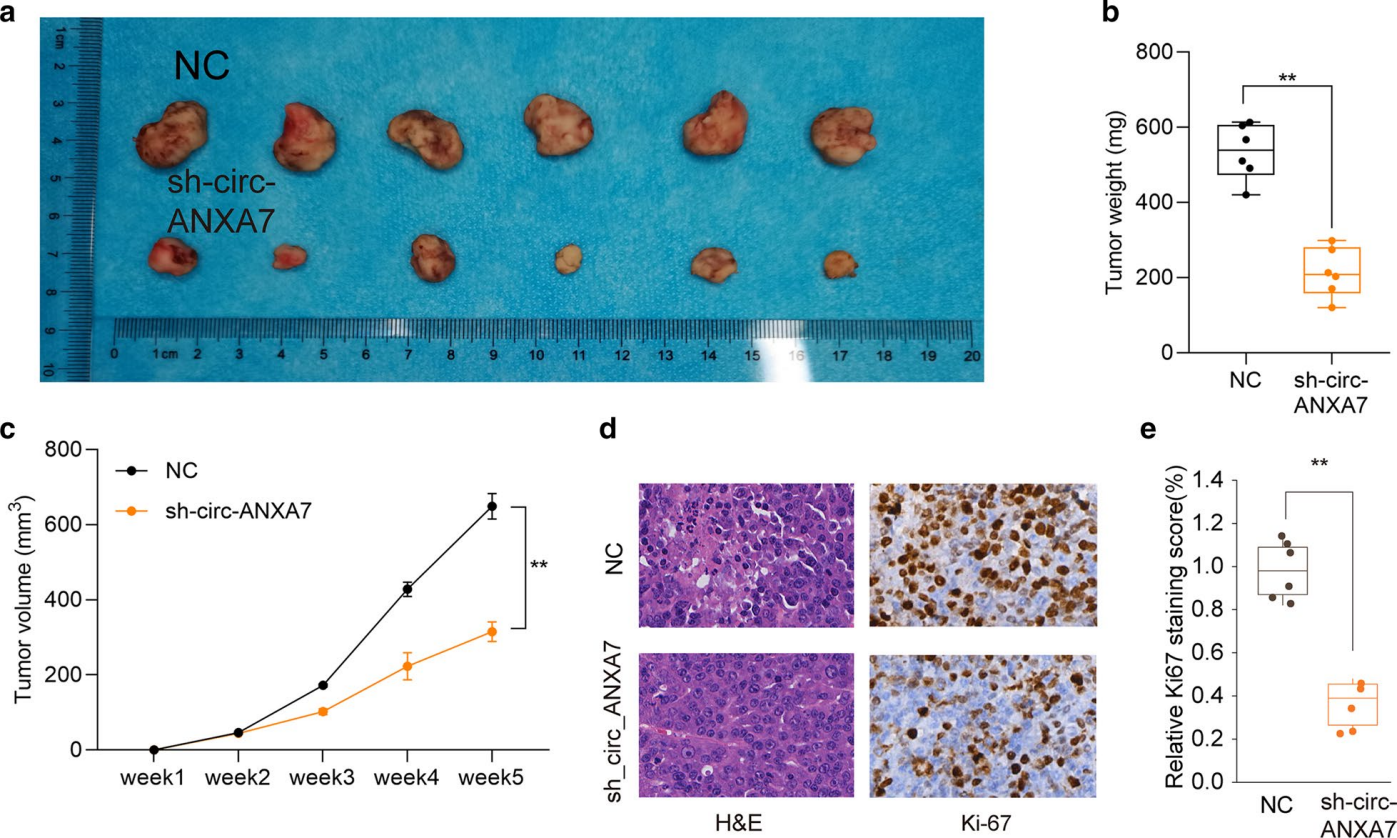
circ-ANXA7 knockdown suppresses tumor growth in vivo for LUAD. **a**, **b** Tumor weight of control and sh-circ-ANXA7 LUAD mice model after 5 weeks. **c** Tumor volume curves in the two groups. **d** Representative images of H&E and immunohistochemistry staining in the mice model after injection with LUAD cells for 5 weeks. **e** Relative Ki67 staining score was quantified in the sh-circ-ANXA7 and control LUAD mice model. **p < 0.01

We investigated the effects of circ-ANXA7 knockdown on tumor growth of LUAD in vivo. Following 5 weeks, the tumor weight was calculated. Compared to the control group, there was significantly lower tumor weight in LUAD model injected by sh-circ-ANXA7 LUAD cells (Fig. 3a, b). Furthermore, sh-circ-ANXA7 distinctly decreased the tumor volume of LUAD mice model (Fig. 3c). H&E and immunohistochemistry results showed that silencing circ-ANXA7 remarkedly suppressed abnormal proliferation of LUAD cells (Fig. 3d, e).

### circ-ANXA7 could directly target the expression of miR-331 in LUAD

**Fig. 4:**
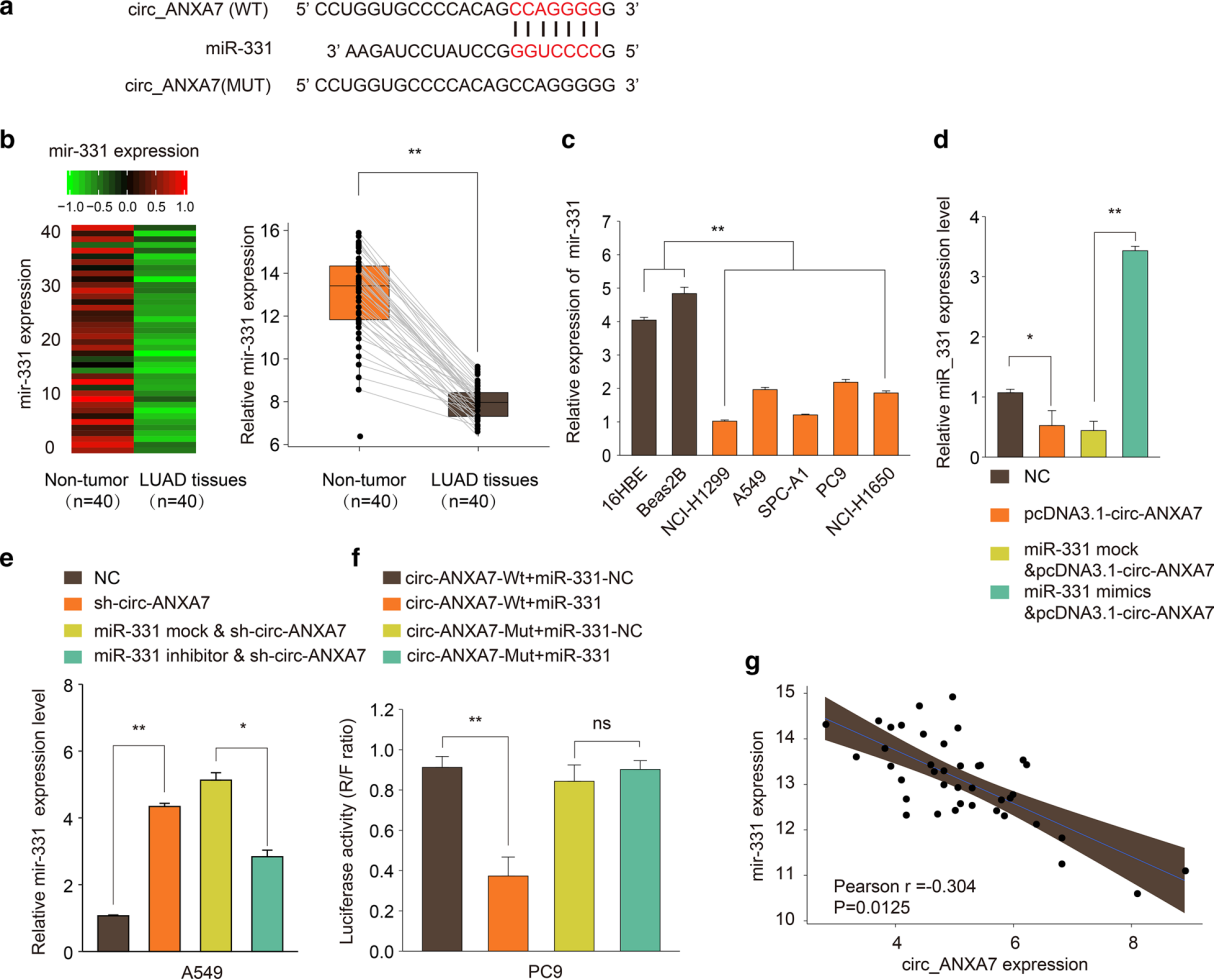
circ-ANXA7 could directly target the expression of miR-331. **a** A schematic diagram showing the putative binding sites between circ-ANXA7 and miR-331. **b** MiRNA microarray expression profiles identified down-regulated miR-331 between LUAD tumor tissues and non-tumor tissues, which was verified by RT-qPCR. **c** Down-regulated miR-331 was determined in LUAD cells compared to controls by RT-qPCR. **d** RT-qPCR was utilized to examine miR-331 expression in A549 cells under transfection of pcDNA3.1-circ-ANXA7 and/or miR-331 mimics. **e** miR-331 expression was determined in A549 cells transfected with sh-circ-ANXA7 and/or miR-331 inhibitor. **f** Dual luciferase report between circ-ANXA7 and miR-331. **g** Correlation between circ-ANXA7 and miR-331. **p < 0.01

After prediction, there were putative binding sites between circ-ANXA7 and miR-331 (Fig. 4a). According to miRNA microarray expression profiles, miR-331 was down-regulated in LUAD tumor tissues compared to non-tumor tissues, which was verified by RT-qPCR (Fig. 4b). In comparison to normal cells, low miR-331 expression was determined in LUAD cells (Fig. 4c). In A549 cells transfected with pcDNA3.1-circ-ANXA7, miR-331 expression was distinctly decreased compared to NC group (Fig. 4d). Decrease in miR-331 expression was ameliorated in LUAD cells following co-transfection with pcDNA3.1-circ-ANXA7 and miR-331 mimics (Fig. 4d). Knockdown of circ-ANXA7 significantly increased miR-331 expression in A549 cells, which was suppressed after co-transfection with sh-circ-ANXA7 and miR-331 inhibitor (Fig. 4e). Dual luciferase report turned out the direct relationship between circ-ANXA7 and miR-331 (Fig. 4f). In a total of 40 LUAD patients, circ-ANXA7 expression was negatively correlated with miR-331 expression (Pearson r = − 0.304 and p = 0.0125; Fig. 4g).

### circ-ANXA7 promotes proliferation and invasion of LUAD cells by targeting miR-331

**Fig. 5:**
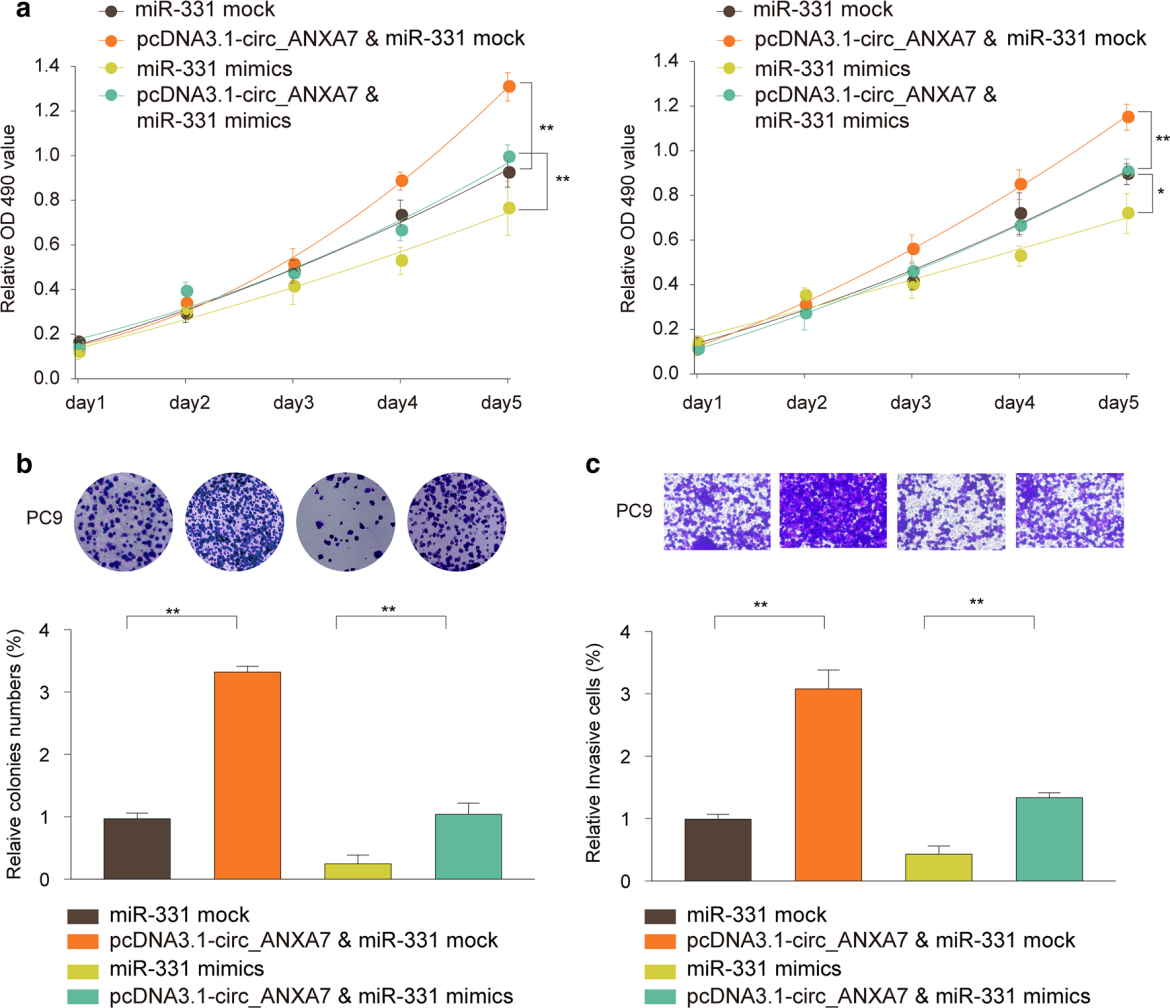
circ-ANXA7 promotes proliferation and invasion of LUAD cells by targeting miR-331. **a** CCK-8 assay was used to detect the cell viability of A549 and PC9 transfected with pcDNA3.1-circ-ANXA7 and/or miR-331 mimics. **b** Proliferative ability of PC9 cells was determined by colony formation assay pcDNA3.1-circ-ANXA7 and/or miR-331 mimics. **c** Transwell assay was utilized to evaluate the invasive capacity of PC9 cells after transfection with pcDNA3.1-circ-ANXA7 and/or miR-331 mimics. **p < 0.01

We further explored the regulatory mechanisms of circ-ANXA7 on biological behavior of LUAD cells. As shown in Fig. 5a, in comparison to controls, miR-331 mimics restrained cell viability of A549 and PC9. Furthermore, cell viability of A549 and PC9 was induced by circ-ANXA7 overexpression, which was suppressed after co-transfection with miR-331 mimics. Colony formation assay results suggested that miR-331 overexpression inhibited proliferation of PC9 cells compared to controls (Fig. 5b). When transfected with pcDNA3.1-circ-ANXA7, proliferative capacity of A549 and PC9 cells was promoted, which was suppressed following co-transfection with miR-331 mimics (Fig. 5b). Compared to controls, miR-331 overexpression markedly inhibited invasive ability of PC9 cells (Fig. 5c). Also, invasive ability of PC9 cells was conspicuously enhanced by pcDNA3.1-circ-ANXA7, which was inhibited after co-transfection with miR-331 mimics.

### LAD1 is a direct target of miR-331 in LUAD

**Fig. 6:**
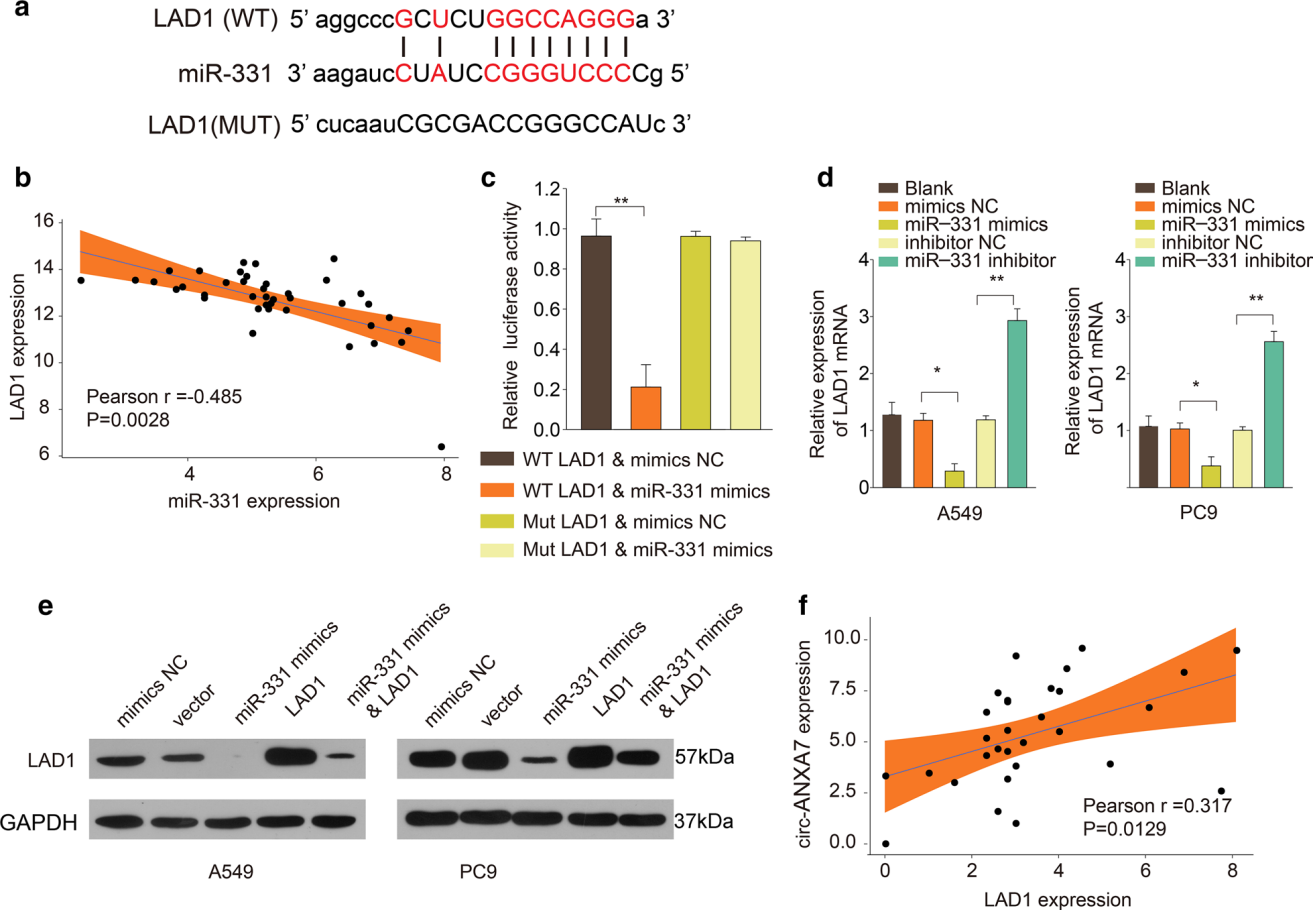
LAD1 is a direct target of miR-331 in LUAD. **a** A schematic diagram depicting the putative binding sites between miR-331 and LAD1. **b** A negative correlation between miR-331 and LAD1 expression. **c** Dual luciferase report verified the interaction between miR-331 and LAD1. **d** LAD1 expression was detected in A549 and PC9 cells transfected with miR-331 mimics or inhibitor using RT-qPCR. **e** Western blot was utilized to examine the expression of LAD1 protein in the two cells transfected by miR-331 mimics and/or LAD1. **f** A positive association between circ-ANXA7 and LAD1 in LUAD. *p < 0.05; **p < 0.01

The putative binding sites between miR-331 and LAD1 were detected, as shown in Fig. 6a. There was a negative correlation between miR-331 and LAD1 in 40 LUAD tissues (Fig. 6b). As dual luciferase report, miR-331 could directly combine with the 3′ UTR of wt-LAD1 (Fig. 6c). Compared to controls, miR-331 mimics prominently decreased the expression of LAD1 mRNA, nevertheless, its inhibitor distinctly increased the expression of LAD1 in A549 and PC9 cells (Fig. 6d). In Fig. 6e, miR-331 mimics significantly decreased the expression of LAD1 protein in the two cells. As shown in Fig. 6f, there was a positive association between circ-ANXA7 and LAD1 in LUAD (Pearson r = 0.317 and p = 0.0129).

### circ-ANXA7 promotes proliferation and invasion of LUAD cells via LAD1

**Fig. 7:**
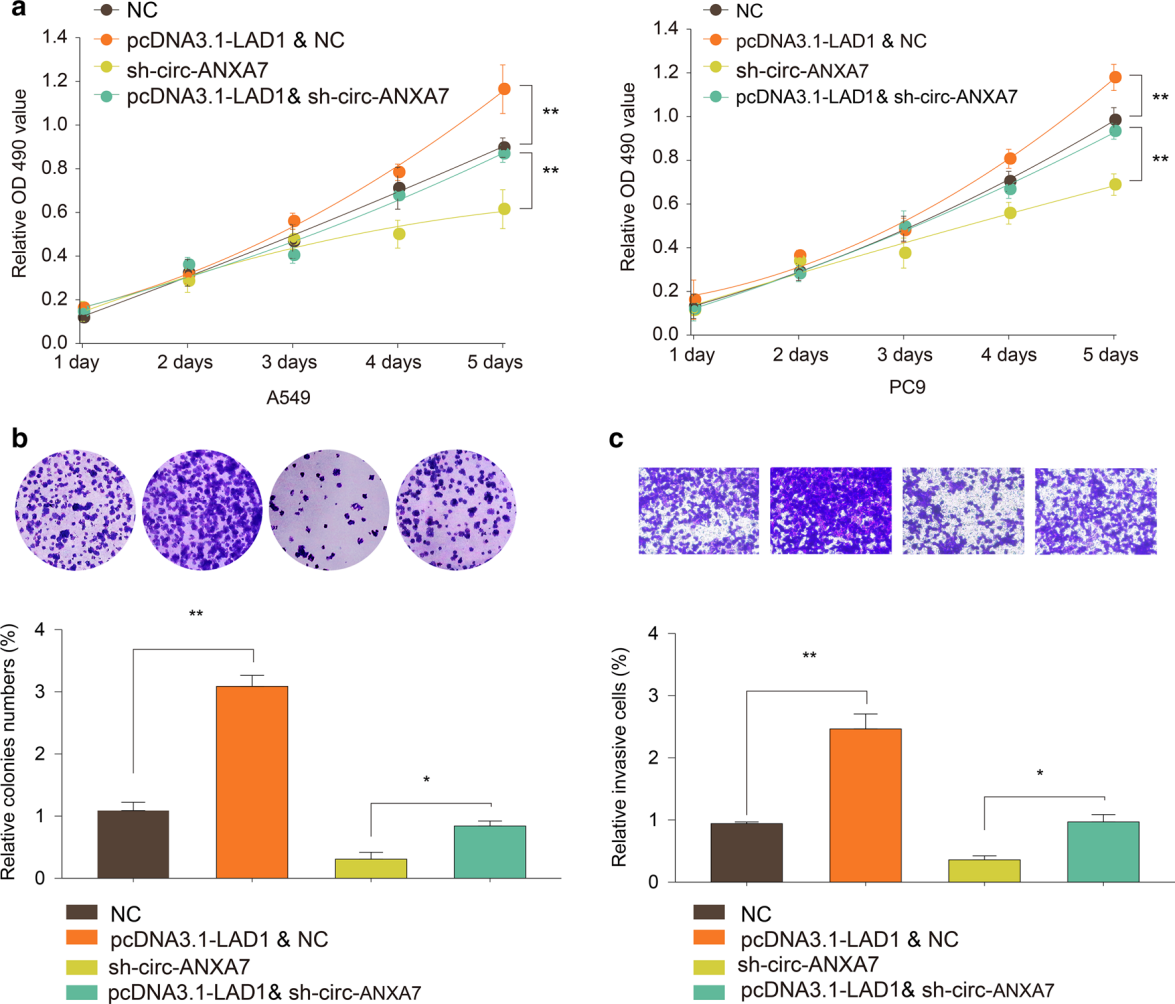
circ-ANXA7 promotes proliferation and invasion of LUAD cells via LAD1. **a** CCK-8 assay was performed to determine cell viability of A549 and PC9 cells transfected with pcDNA3.1-LAD1 and/or sh-circ-ANXA7. **b** Colony formation assay was utilized to examine the proliferative ability of PC9 cells transfected with pcDNA3.1-LAD1 and/or sh-circ-ANXA7. **c** The invasive capacity of PC9 cells was detected following transfection with pcDNA3.1-LAD1 and/or sh-circ-ANXA7. *p < 0.05; **p < 0.01

CCK-8 results revealed that LAD1 overexpression facilitated cell viability of A549 and PC9 cells (Fig. 7a). However, when co-transfected with pcDNA3.1-LAD1 and sh-circ-ANXA7, cell viability of the two cells was significantly inhibited. In Fig. 7b, proliferative ability of PC9 cells was remarkably promoted by transfection of pcDNA3.1-LAD1. When transfected with sh-circ-ANXA7, cell proliferative ability was suppressed, which was improved following co-transfection with pcDNA3.1-LAD1. Furthermore, transwell assay results demonstrated that pcDNA3.1-LAD1 significantly elevated the invasive capacity of PC9 cells (Fig. 7c). In comparison to sh-circ-ANXA7, invasion ability of PC9 cells was distinctly elevated after co-transfection with pcDNA3.1-LAD1.

### LAD1 is a prognostic marker for LUAD

**Fig. 8:**
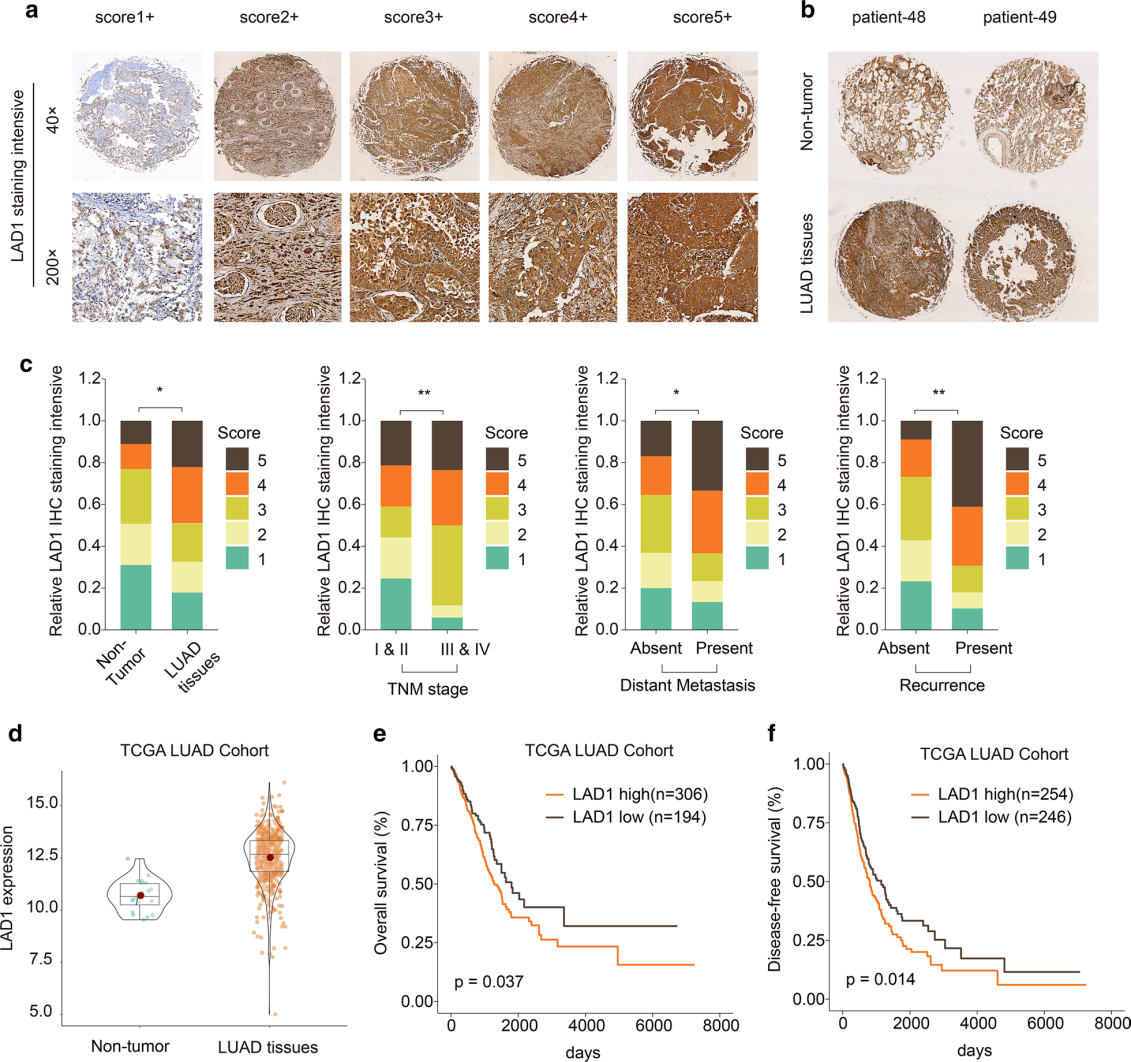
LAD1 is a prognostic marker for LUAD. **a** LAD1 immunohistochemistry staining intensity of LAD1 according to its scores. Magnification: ×40; ×200. **b** Immunohistochemistry of LAD1 between non-tumor and LUAD tissues. **c** Proportion of LAD1 immunohistochemistry staining intensity in TNM stage III & IV vs. I & II, in present distant metastasis vs. absent distant metastasis, in present recurrence vs. absent recurrence. **d** High LAD1 expression in LUAD tissues compared to non-tumor tissues among TCGA-LUAD cohort. **e**, **f** Overall survival and disease-free survival analysis of LAD1 expression for LUAD patients. *p < 0.05; **p < 0.01

From Human Protein Atlas database, IHC of LAD1 was obtained. In Fig. 8a, as the score increased, the staining intensity of LAD1 gradually increased. Compared to non-tumor tissues, its staining intensity was stronger in LUAD tissues (Fig. 8b). In comparison to LUAD tissues, high IHC staining scores occupied a higher proportion (Fig. 8c). Furthermore, there was a higher proportion of high IHC staining scores in TNM stage III & IV vs. I & II, in present distant metastasis vs. absent distant metastasis, in present recurrence vs. absent recurrence (Fig. 8c). Based on TCGA database, we found that the expression pattern of LAD1 was different in different cancers (Additional file 1: Fig. S1). In TCGA-LUAD cohort, LAD1 expression was significantly higher in LUAD tissues in comparison to non-tumor tissues (Fig. 8d). Its high expression usually predicted a poorer overall survival and disease-free survival (Fig. 8e, f). Furthermore, in patients with TNM III & IV, high LAD1 expression predicted a worsen overall survival and disease-free survival (Additional file 2: Fig. S2). In Table 1, LAD1 expression was significantly correlated with tumor size, lymph metastasis and recurrence status. After univariate and multivariate analysis, LAD1 expression could become a potential prognostic marker for NSCLC (Table 2).

**Table 1:** Correlation of clinic-pathological features with LAD1 expression in NSCLC TMA cohort

Variables	Clinicopathological features	LAD1 expression		
		Low expression (n = 53)	High expression (n = 42)	p-value
Age (years)	≤ 65	23 (43.4%)	23 (54.8%)	0.371
	> 65	30 (56.6%)	19 (45.2%)	
Gender	Male	35 (66.0%)	23 (54.8%)	0.364
	Female	18 (34.0%)	19 (45.2%)	
Smoking history	Smokers	38 (71.7%)	24 (57.1%)	0.207
	Never smokers	15 (28.3%)	18 (42.9%)	
Histologic subtype	Squamous cell carcinoma	17 (32.1%)	20 (47.6%)	0.183
	Adenocarcinoma	36 (67.9%)	22 (52.4%)	
TNM stage	Stage I & II	44 (83.0%)	26 (61.9%)	0.020
	Stage III	9 (16.9%)	16 (38.0%)	
Tumor size (cm)	≤ 5	45 (84.9%)	25 (59.5%)	0.001***
	> 5	8 (15.1%)	17 (40.5%)	
Lymph metastasis	No	42 (79.2%)	23 (54.8%)	0.02*
	Yes	11 (20.8%)	19 (45.2%)	
Recurrence status	No	41 (77.4 %)	15 (35.7 %)	< 0.0001****
	Yes	12 (22.6 %)	27 (64.3 %)	

*p < 0.05; ***p < 0.001; ****p < 0.0001

**Table 2:** Univariate and multivariate analysis of clinic-pathological features and LAD1 expression in NSCLC TMA cohort (n = 95)

	Univariate analysis			Multivariate analysis		
	HR	95% CI	P value	HR	95% CI	p value
Age	0.985	0.759–1.496	0.659			
Gender	0.645	0.452–1.228	0.598			
Smoking history	1.269	0.884–1.549	0.195			
Histologic subtype	0.799	0.224 − 0.120	0.344			
TNM stage	3.466	2.359–4.988	0.001	2.498	1.701–3.273	0.009**
Tumor size(cm)	2.215	1.498–2.970	0.059	1.765	0.921–2.431	0.198
Lymph metastasis	2.278	1.959–2.995	0.040	1.745	1.207–2.301	0.039*
Recurrence status	3.478	2.214–4.987	0.002	3.048	2.364–3.699	0.001***
LAD1 expression	2.639	1.759–3.648	0.019	2.455	1.904–2.959	0.025*

*p < 0.05; ***p < 0.001; ****p < 0.0001

## Discussion

LUAD is one of the most common malignancies worldwide [4, 20–22]. Due to the lack of effective diagnosis and treatment, its treatment effect is still unsatisfactory [23–25]. circRNA is a novel non-coding RNA. Many studies have shown that circRNA plays a key role in the tumor progression [11, 26, 27]. In our research, we found that circ-ANXA7 was significantly increased in LUAD tissues and cells, and may have potential as a biomarker for the prognosis and prognosis of LUAD. At present, no research has reported the role of this circRNA in tumors. Through functional analysis, circ-ANXA7 can act as a sponge for miR-331, thereby alleviating the inhibitory effect of this miRNA on its target gene LAD1 in LUAD cells.

A series of cellular experiments demonstrated that knockdown of circ-ANXA7 remarkably suppressed viability, proliferation, migration and invasion, and induced apoptosis for LUAD cells. More importantly, in vivo, silencing circ-ANXA7 distinctly inhibited tumor growth. These findings revealed that circ-ANXA7 could be involved in the progression of LUAD. As previous research, several circRNAs have been identified for LUAD. For example, circ-SOX4 can promote the development of LUAD and activate the WNT signaling pathway by stimulating miR-1270/PLAGL2 [28].

Our data suggested that there was a negative correlation between circ-ANXA7 and miR-331 expression in LUAD tissues. Dual luciferase report confirmed the direct relationship between the two. Thus, circ-ANXA7 could become a sponge of miR-331 in LUAD. miR-331 was down-regulated in LUAD tissues and cells, consistent with a previous study [29]. miR-331 could be in association with LUAD prognosis [29]. Circulating miR-331-3p possesses underlying potential for early diagnosing and supervising of lung cancer [30]. miR-331-3p has been identified to be down-regulated in NSCLC, which could be an independent prognostic factor [16]. miR-331-3p could distinctly suppress invasion and metastasis of NSCLC cells. Furthermore, miR-331-3p could be sponged by circ-0001649 in NSCLC [31]. Consistently, our results demonstrated that miR-331 overexpression inhibited proliferation as well as invasion for LUAD cells.

In this research, high LAD1 expression was detected both in LUAD cells and tissues. LAD1 up-regulation conspicuously accelerated cell viability, proliferation, and invasion of LUAD cells. Dual luciferase report analysis confirmed the target relationship between miR-331 and LAD1. Furthermore, LAD1 expression was in significant association with TNM stage, distant metastasis, and recurrence for LUAD patients. Its high expression indicated poorer overall survival and disease-free survival time. In line with univariate and multivariate regression analyses, LAD1 expression could be an independent prognostic factor for LUAD after adjusting other clinical indicators. As previous studies, LAD1 mediates the proliferation and migration of breast cancer cells [32]. Moreover, LAD1 promoter methylation is a promising prognostic marker for renal cell carcinoma [33]. In-depth research requires to be carried out to validate the function of LAD1 in LUAD.

In this study, we found an up-regulated circRNA circ-ANXA7 in LUAD. Further studies verified that circ-ANXA7 could induce proliferation and invasion of LUAD cells and promote tumor growth. Mechanistically, circ-ANXA7 can mediate the progression of LUAD via miR-331/LAD1. Our study could provide a valuable theoretical basis for exploring the potential therapeutic targets of LUAD.

## Conclusions

Our findings identified a novel circRNA circ-ANXA7, which was up-regulated both in LUAD cells and tissues. As a sponge of miR-331, it induced proliferation and invasion of LUAD cells by promotion of LAD1. Besides, circ-ANXA7 knockdown suppressed tumor growth. Its low expression indicated a poor prognosis of LUAD patients. These findings demonstrated the function and underlying mechanism of circ-ANXA7 in LUAD. Larger cohort studies are needed to clarify the prognostic potential of circ-ANXA7 in LUAD.

## Supplementary Information

**Additional file 1: Fig. S1.** Box plots showing the expression patterns of LAD1 in different types of cancers.

**Additional file 2: Fig. S2.** Overall survival and disease-free survival analysis of LAD1 for LUAD patients with TNM stage I & II or III & IV.

## Data Availability

The datasets used and/or analysed during the current study are available from the corresponding author on reasonable request.

## Abbreviations

LUAD: Lung adenocarcinoma
circRNAs: Circular RNAs
NSCLC: Non-small cell lung cancer
LUSC: Lung squamous cell carcinoma
qRT-PCR: Quantitative reverse transcription PCR
NC: Negative control
EdU: 5-Ethynyl-2′-deoxyuridine
IVCs: Independent ventilation cages
H&E: Haematoxylin and eosin
