# Supplementary figures and images for: circ-ANXA7 facilitates lung adenocarcinoma progression via miR-331/LAD1 axis
Source: Cancer Cell Int. 2021 Feb 3;21:85. doi: 10.1186/s12935-021-01791-5 (PMC7860208; doi:10.1186/s12935-021-01791-5)

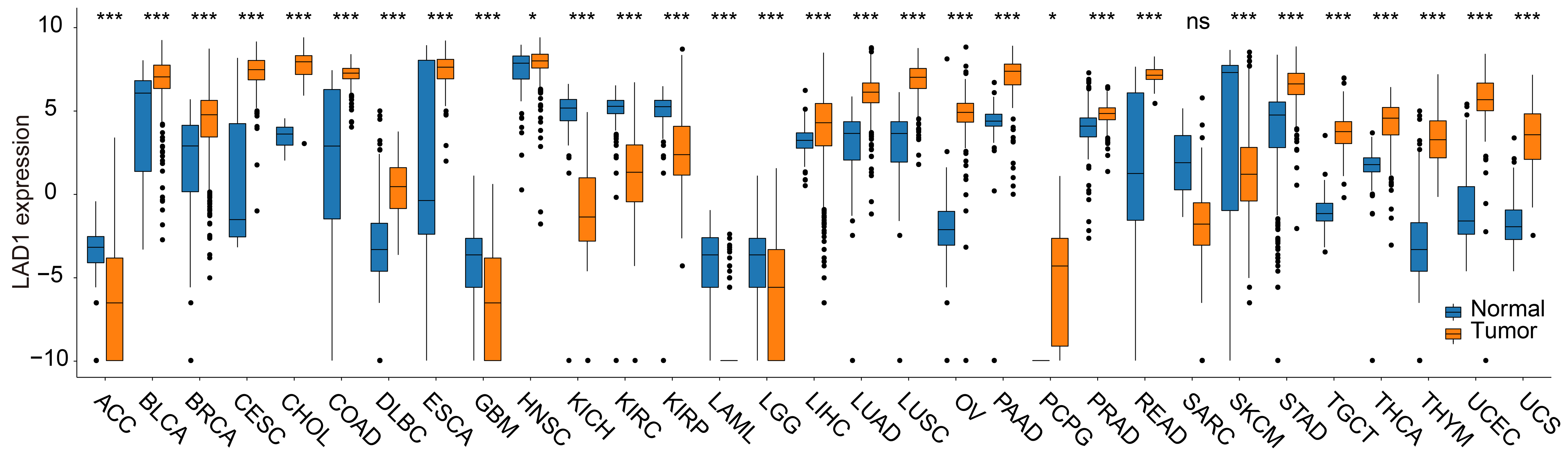

Supplement: Supplementary file 1 — Additional file 1: Fig. S1. Box plots showing the expression patterns of LAD1 in different types of cancers. [file 12935_2021_1791_MOESM1_ESM.tif]

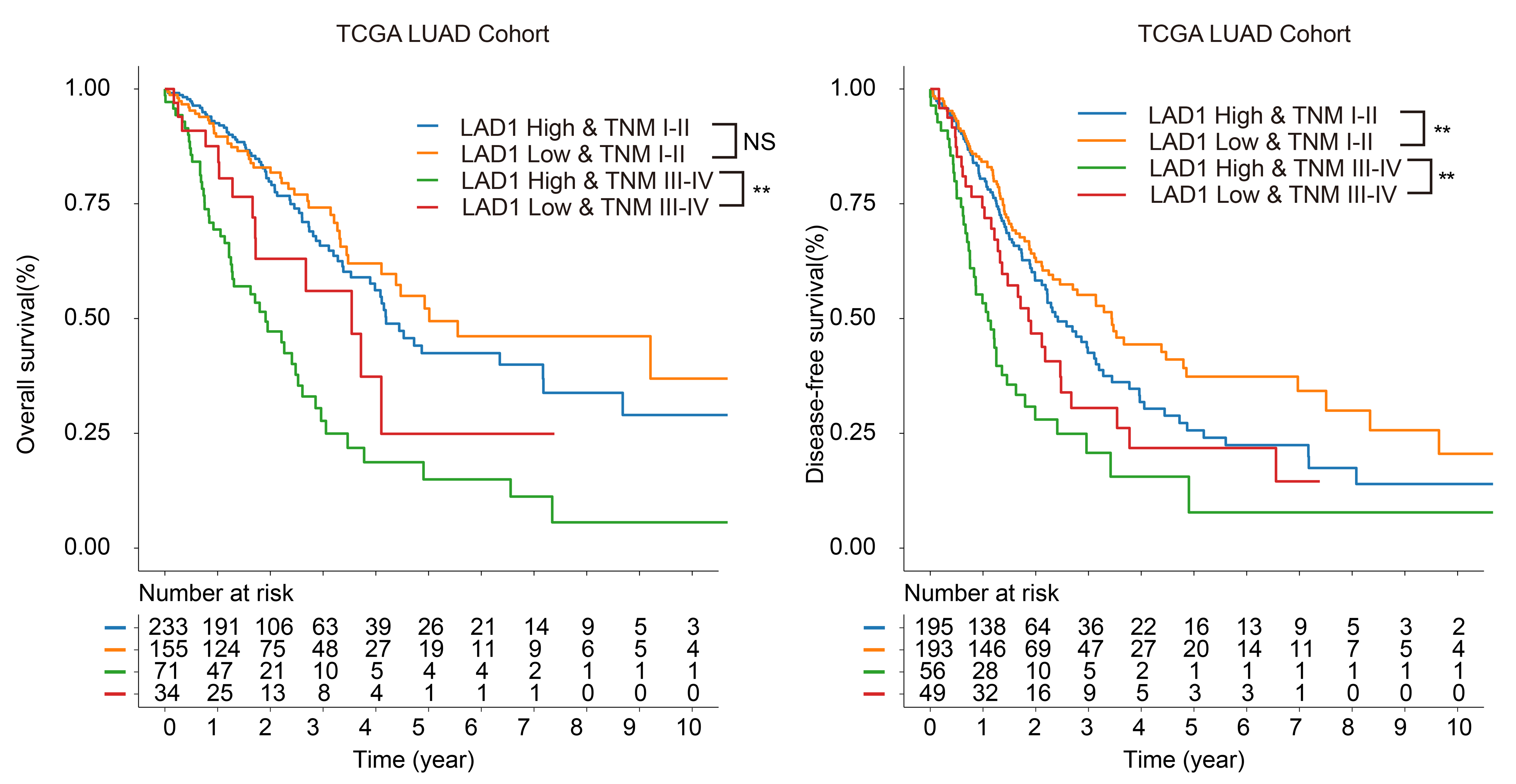

Supplement: Supplementary file 2 — Additional file 2: Fig. S2. Overall survival and disease-free survival analysis of LAD1 for LUAD patients with TNM stage I & II or III & IV. [file 12935_2021_1791_MOESM2_ESM.tif]
